# Supplementary material for: Identification of photoperiod-induced specific miRNAs in the adrenal glands of Sunite sheep (Ovis aries)
Source: Front Vet Sci. 2022 Jul 22;9:888207. doi: 10.3389/fvets.2022.888207 (PMC9354845; doi:10.3389/fvets.2022.888207)
Supplement: Supplementary file 1 [file Data_Sheet_1.ZIP › Supplementary file/miRDeep2 file.zip!/miRDeep2 file/Novel_Structure/SPLP42A1/Novel_483.pdf]

Provisional ID	:	NC_019476.2_25147
Score total	:	110.9
Score for star read(s)	:	3.9
Score for read counts	:	103.1
Score for mfe	:	2.2
Score for randfold	:	1.6
Score for cons. seed	:	
Total read count	:	214
Mature read count	:	211
Loop read count	:	0
Star read count	:	3

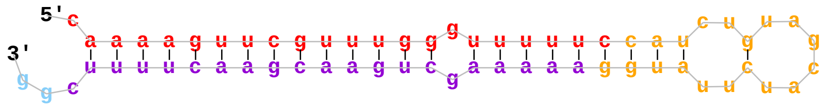

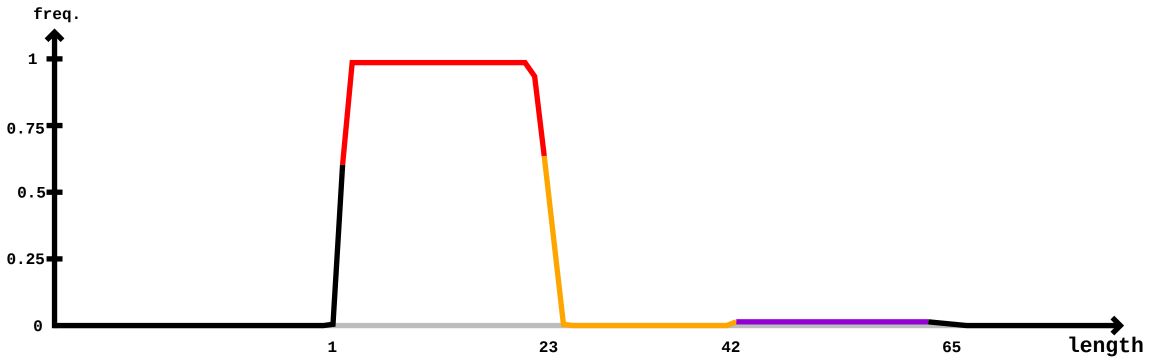

Mature **Star**

```
5'- guccacaagucuuuguauuagguuggcccaaaaguucguuuggguuuuuccaucuguagcaucuuauggaaaaagcugaacgaacuuuucggccaauccaguaugagga   -3'   obs
    guccacaagucuuuguauuagguuggcccaaaaguucguuuggguuuuuccaucuguagcaucuuauggaaaaagcugaacgaacuuuucggccaauccaguaugagga         exp
    .........((((((((((.((((((((.((((((((((((((.(((((((((..(......)..))))))))).))))))))))))))).)))))))).))))))))))    reads   mm   sample
    ...........................ccaaaaguucguuuggguuuuu............................................................    1       0    seq
    ............................Aaaaaguucguuuggguuuuu............................................................    62      1    seq
    ............................Aaaaaguucguuuggguuuuuc...........................................................    66      1    seq
    .............................aaaaguucguuuggguuuu.............................................................    10      0    seq
    .............................aaaaguucguuuggguuuC.............................................................    1       1    seq
    .............................aaaaguucguuuggguuuuG............................................................    1       1    seq
    .............................aaaGguucguuuggguuuuuc...........................................................    1       1    seq
    .............................aaaaguucguuuggguuuuuA...........................................................    3       1    seq
    .............................aaGaguucguuuggguuuuuc...........................................................    2       1    seq
    .............................aaaaguucguuuggguuuuucG..........................................................    7       1    seq
    .............................aaaaguucguuuggguuuuucA..........................................................    56      1    seq
    .............................aaaaguucguuuggguuuuucAa.........................................................    1       1    seq
    .....................................................................aaaaaCcugaacgaacuuuuc...................    3       1    seq
```
